# End User Participation in the Development of an Ecological Momentary Intervention to Improve Coping With Cannabis Cravings: Formative Study

**DOI:** 10.2196/40139

**Published:** 2022-12-15

**Authors:** Molly A Anderson, Alan J Budney, Nicholas C Jacobson, Inbal Nahum-Shani, Catherine Stanger

**Affiliations:** 1 Center for Technology and Behavioral Health Geisel School of Medicine Dartmouth College Lebanon, NH United States; 2 Institute for Social Research University of Michigan Ann Arbor, MI United States

**Keywords:** cannabis, formative, distraction, mindfulness, coping, youth, public health, mental health, health intervention, ecological momentary intervention

## Abstract

**Background:**

Cannabis misuse in young adults is a major public health concern. An important predictor of continued use is cannabis craving. Due to the time-varying nature of cravings, brief momentary interventions delivered while cravings are elevated may improve the use of strategies to cope with cravings and reduce cannabis use.

**Objective:**

The goal of this manuscript is to describe a formative study to develop coping strategy messages for use in a subsequent intervention.

**Methods:**

Young adults (aged 19-25 years; n=20) who reported using cannabis >10 of the past 30 days recruited via social media participated in this formative study. Participants rated an initial set of 15 mindfulness and 15 distraction coping strategies on a scale from 1 to 4 (very low degree to very high degree) for clarity, usefulness, and tone. They also provided comments about the content.

**Results:**

Participants found the initial distraction messages slightly clearer than mindfulness (mean 3.5, SD 0.4 and mean 3.4, SD 0.4, respectively), both were comparable in tone (mean 3.2, SD 0.5 and mean 3.2, SD 0.4, respectively), and mindfulness messages were more useful than distraction (mean 3.0, SD 0.5 and mean 2.8, SD 0.6, respectively). Of the 30 messages, 29 received a rating of very low or low (<2) on any domain by >3 participants or received a comment suggesting a change. We revised all these messages based on this feedback, and the participants rated the revised messages approximately 2 weeks later. Participants earned US $10 for completing the first and US $20 for the second survey. The ratings improved on usefulness (especially the distraction items) with very little change in clarity and tone. The top 10 messages of each coping type (mindfulness and distraction) were identified by overall average rating (collapsed across all 3 dimensions: all rated >3.0). The final items were comparable in clarity (distraction mean 3.6, SD 0.4; mindfulness mean 3.6, SD 0.4), tone (distraction mean 3.4, SD 0.4; mindfulness mean 3.4, SD 0.4), and usefulness (distraction mean 3.1, SD 0.5; mindfulness mean 3.2, SD 0.5).

**Conclusions:**

The inclusion of end users in the formative process of developing these messages was valuable and resulted in improvements to the content of the messages. The majority of the messages were changed in some way including the removal of potentially triggering language. These messages were subsequently used in an ecological momentary intervention.

## Introduction

Young adults between the ages of 19 and 25 years are in a time of major life changes during which they are developing new social relationships, experiencing increased independence, and developing the skills necessary to regularly make healthy choices [1]. This time of transition is a period during which additional support for healthy choices is critical, including refraining from substance misuse. Cannabis use among young adults in the United States has increased considerably over the past few decades, while the perceived risks of using cannabis have decreased [2]. In 2020, an estimated 13.5% of young adults aged 18-25 years met the criteria for cannabis use disorder [3]. Greater quantity and frequency of cannabis use are associated with increased cannabis-related problems, such as increased psychological distress, loneliness, and detrimental effects on memory [4-9].

Craving is one predictor of subsequent cannabis use and may be an important target for intervention when attempting to reduce or quit use [10,11]. The term “craving” has a history of slightly different definitions [12]; however, most definitions have in common that they refer to the desire for the drug or desire to experience the resulting effects of using the drug. Some definitions distinguish between “cravings” and “urges,” suggesting that the term “craving” be restricted to referencing a desire for the effects of using a drug, whereas the term “urge” be used when referencing intent to use a drug [13]. Some definitions conceptualize “cravings” and “urges” as the same phenomenon at different points on a spectrum with “craving” used to indicate an extreme desire and “urge,” indicating a lesser desire [14,15]. Although there have been arguments suggesting distinct definitions, individuals respond similarly to items assessing “cravings” and “urges” [16]. Here, we do not distinguish between cannabis cravings and the urge to use cannabis.

Mindfulness and distraction are two strategies to reduce cravings [17]. These two strategies have distinct theoretical bases: mindfulness involves maintaining attention on an immediate experience while adopting an accepting and curious perspective, whereas distraction involves active engagement with an alternative activity to direct focus away from the craving experience [17,18]. Mindfulness has been shown to be a useful strategy to cope with cravings and prevent relapse following periods of abstinence from cigarette and alcohol use [19-21]. More relevantly, the implementation of a mindfulness practice reduces the relationship between cannabis cravings and subsequent use [22,23]. Support for distraction as a coping strategy is mixed; some studies suggest distraction may be maladaptive [24], and others suggest there may be no relationship between distraction as a coping mechanism and craving [10]. However, other research shows distraction to be an effective coping mechanism, even outperforming mindfulness as a strategy to cope with cravings [25].

Direct comparisons of mindfulness and distraction coping strategies have largely been limited to controlled laboratory settings, limiting the generalizability of these findings. The extent to which mindfulness or distraction are effective as coping mechanisms likely depends on the environmental context at a given moment [26,27]. Additionally, though many therapeutic programs teach strategies for coping with cravings [18,28], these require individuals to learn a strategy at a time when cravings may not be present or distressing, then implement the strategy later when they are experiencing uncomfortable or distressing levels of craving. Because craving levels vary throughout the day, it may be beneficial to provide support when craving levels are high. Digital interventions can provide such time-varying support.

Several app-based interventions have shown success in helping people who use cannabis reduce their use [29,30]. Participants generally found these apps to be acceptable as an intervention for cannabis use. Although these mobile interventions provided support to individuals attempting to reduce their cannabis use—including strategies for coping with cannabis cravings—they did not necessarily provide support at the moment when the need is high (eg, when the urge to use is high). Rather, these apps mimic the structure of traditional therapy wherein participants engage with psychoeducational content and develop important skills but do not assess momentary craving or push momentary support when the need is high. However, digital interventions have the capability of providing momentary support in the form of advice, information, or coping strategies recommended to the participant via text (eg, using SMS text messages or within an app). Such interventions have shown promising results in providing support to individuals who are trying to quit smoking [31,32], and in reducing risky alcohol use [33]. The inexpensive and brief nature of interventions that rely on communication via text or direct messages allows for greater flexibility in intervention timing. This can allow for the delivery of a message when a participant is in great need of support, such as when a participant is in proximity to trigger locations detected via passive sensing [34], when the participant texts requesting support [32], or when elevated need is identified by periodic ecological momentary assessment (EMA). Currently, there are no such interventions to help young adults cope with cravings as they reduce their cannabis use.

An ongoing concern with digital interventions is the promotion of engagement with the intervention [35]. Although digital interventions may reduce barriers to accessing treatment [36], the target individuals still must engage with (ie, invest physical, emotional, and cognitive energy in [37]) the intervention to see positive effects. One way to improve the success of a digital intervention is through user-centered design to include the target population in the formative stages of the intervention [38]. The purpose of this study was to develop messages containing mindfulness- or distraction-based coping strategies for use in an ecological momentary intervention to help young adults cope with cannabis cravings as they try to reduce their cannabis use.

## Methods

### Participants

Participants included 20 young adults (19-25 years; mean 21.65, SD 1.79 years; n=9, 45% male; n=9, 40% non-White or Hispanic; see Table 1 for demographic details) who responded to a Facebook ad recruiting people who use cannabis for a research study and were interested in reducing their use and reported using cannabis ≥10 out of the past 30 days. Individuals who were pregnant or breastfeeding or who reported being in treatment for problems related to substance use were excluded from participation. These inclusion and exclusion criteria were selected to ensure consistency with the eligibility criteria for the later trial of the ecological momentary intervention for which the messages were being developed.

**Table 1:** Demographic characteristics of participants.

Participant characteristics		Values, n (%)
**Gender**		
	Female	9 (45)
	Male	9 (45)
	Nonbinary	2 (10)
**Race**		
	White	14 (70)
	Black or African American	1 (5)
	Asian	2 (10)
	American Indian or Alaska Native	1 (5)
	Other (not specified)	2 (10)
**Ethnicity**		
	Hispanic or Latino	3 (15)
	Not Hispanic or Latino	17 (85)

### Ethical Considerations

All procedures were reviewed and approved by the institutional review board at Dartmouth College (#32248). Participants clicked on a link in a Facebook ad and were brought to an online survey where they received general information about the study and were asked if they consented to be screened for eligibility to participate. All consent procedures were embedded in questions within the survey. Prospective participants who affirmatively consented to be screened were asked questions to determine their eligibility for participation. Those who met the inclusion criteria were presented with more detailed information about the study and asked if they consented to participate in the study. Affirmative consent was required before progressing to participate in the study survey.

### Procedures

After completing the eligibility screening and providing informed consent, the participants completed a survey to evaluate a bank of 30 messages consisting of mindfulness-based or distraction-based suggestions for how to cope with cannabis cravings. The initial message items are in Multimedia Appendices 1 and 2. The initial bank of 15 mindfulness messages were adapted from Witkiewitz et al [33] and Spears et al [39]. These studies used mindfulness messages in mobile interventions with the aim of reducing alcohol use and smoking. The initial bank of 15 distraction messages was adapted from Guarino et al [40], which tested a web-based intervention for self-management of pain in individuals who have problems managing their opioid medications. We adapted the messages used in these sources to apply to coping with cannabis cravings, primarily by replacing any references to alcohol use, smoking, or opioid use with references to cannabis use. These strategies and messages could be adapted to be relevant to behaviors in addition to cannabis use by adjusting the wording such that it applies to other substances.

The goal of this study was to select a final bank of 20 total messages (10 mindfulness and 10 distraction) for use in an ecological momentary intervention that would present 1 randomly selected message from the bank of 20 messages when participants reported an urge to use cannabis ≥4 on a scale of 0-10. The number of messages was selected to prevent habituation and boredom by ensuring that a variety of coping strategies are delivered to the participants across a 4-week intervention. All participants in this study were given the same 30 messages at the same time to provide their ratings. Beginning with a bank of 30 possible messages allowed for the 10 lowest-rated messages to be removed from the final message bank.

We implemented a rating scale used in other formative research that aimed to develop a text-based intervention to reduce alcohol use among college students [41]. Participants rated messages on a scale from 1 to 4 (very low degree to very high degree) for understanding (this message is easy to understand), usability (this message is useful), and tone (this message has a good overall tone [41]). We also asked participants to provide any comments they had about how to improve each message in a free response box. Following the first round of feedback, we reviewed all messages and revised any messages that received a rating of very low or low on any of the domains by at least 3 participants. After revising the messages, we sent a second survey (approximately 2 weeks after completing the first survey) asking participants to reevaluate the messages using the same scale and criteria. Participants were compensated US $10 for completing the first survey and US $20 for completing the second survey. The top 10 messages in each category (mindfulness and distraction) were selected for use in the ecological momentary intervention.

## Results

### Initial Quantitative Results

**Figure 1:**
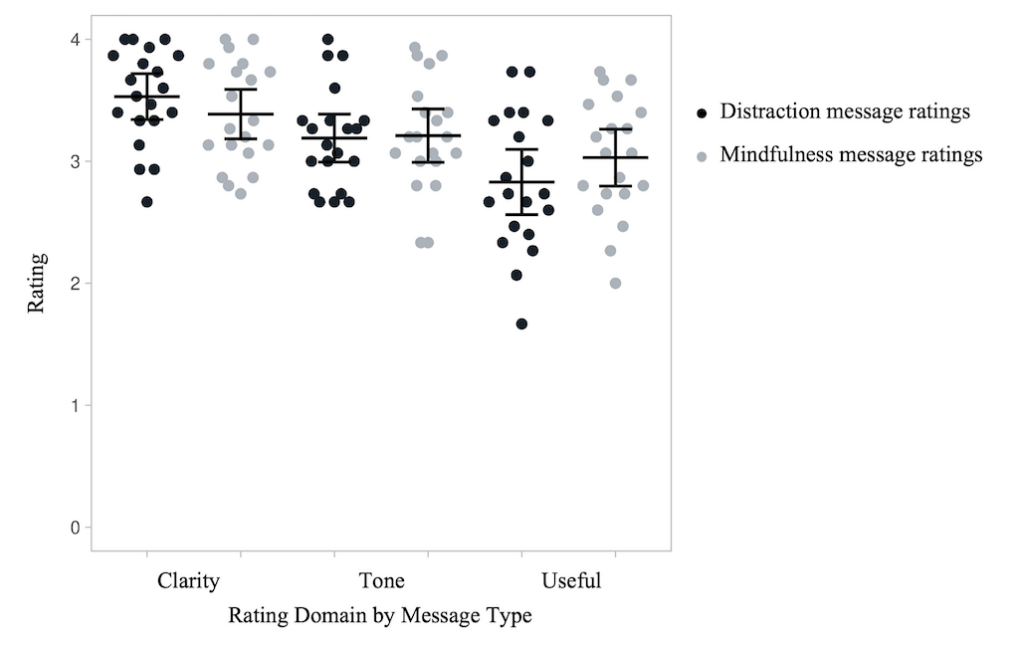
Initial participant ratings (n=20) of the distraction and mindfulness messages. Each data point shows one participant’s average rating of the 15 distraction or 15 mindfulness messages on each of the three domains (clarity, tone, and usefulness). Horizontal bars show average ratings and error bars are 95% CIs.

Figure 1 shows the mean ratings for each category and message type following the first round of ratings. Table 2 shows the mean ratings for each category and message type at both timepoints. The participants found the initial distraction messages more clear than the mindfulness messages (t_19_=2.64, *P*=.02), both were comparable in tone (t_19_=0.28, *P*=.78), and mindfulness messages more useful than distraction (t_19_=2.36, *P*=.03).

**Table 2:** Mean (SD) and range of message ratings.

Timepoint		Distraction			Mindfulness		
		Clear	Tone	Useful	Clear	Tone	Useful
**1 (N=20)**							
	Mean (SD)	3.53 (0.39)	3.21 (0.46)	2.83 (0.56)	3.39 (0.42)	3.19 (0.41)	3.03 (0.49)
	Range	2.67-4.00	2.33-3.93	1.67-3.73	2.73-4.00	2.67-4.00	2.00-3.73
**2 (n=18)**							
	Mean (SD)	3.50 (0.44)	3.27 (0.51)	2.96 (0.48)	3.49 (0.44)	3.32 (0.46)	3.07 (0.54)
	Range	2.67-4.00	2.47-4.00	2.20-4.00	2.67-4.00	2.20-3.93	2.00-3.93

### Initial Qualitative Results

Major feedback themes for the mindfulness messages included concerns about the clarity of the messages, suggestions for rewording the message content, concerns about the suggestion being too difficult to implement at the time cravings were high, and positive responses to the nonjudgmental nature of the messages. Major feedback themes for the distraction messages included confusion about the rationale of the message and how it relates to coping with cannabis cravings, indications that the strategy would not be helpful to them, and concerns that the strategy could be triggering (eg, use of social media as a distraction technique could bring up images that are related to cannabis use). Textbox 1 shows feedback categories and example quotes from participants. The first author identified the feedback categories by finding common themes across participant comments. The first author and a research assistant each independently categorized all feedback statements with 82% agreement. Out of the 30 messages we asked the participants to rate, 29 messages received a rating of very low or low (≤2) on at least 1 of the 3 domains by ≥3 participants or received a comment suggesting a change to the message. Although the sample size is relatively small for this study, we kept the threshold for revising items low with the intention of creating the best possible messages for our subsequent intervention. The lead and senior authors (MAA and CS) reviewed these messages, the quantitative ratings, and the qualitative feedback and revised the messages based on both the domains that were rated low and the open-ended feedback the participants provided. For example, if a message received a low rating on “tone,” we revised the language of the message to make the message friendlier, more empathetic, and more encouraging while maintaining the general strategy the message provided (see Textbox 2 for an example). We implemented participants’ suggestions about how to reword messages whenever possible.

**Examples of participant feedback. The first author found common themes across participant feedback comments which resulted in these feedback categories. This textbox shows the example of participant feedback for each category.**

**Reword**

- Last sentence could reverse: “you don't need to act on any urges you may feel to use cannabis”

**Clarity**

- Had to read over a second time to understand the message

**Too difficult to implement**

- This may be true, but it's very hard to implement in real life. Message doesn't provide a good strategy to use this method IMO

**Nonjudgmental**

- This perspective feels empathic and helpful

**Rationale**

- But why? What's the benefit

**Triggering**

- As I said before, music tends to be more enjoyable when high. I think it is still effective, but it also may make the person want to use cannabis before zeroing in on his or her favorite song.

**Not helpful**

- Vague and not particularly helpful.

**Example of message revision. The lead and senior authors used the quantitative ratings and qualitative feedback to revise the messages. This textbox shows an example of how a message was revised using qualitative feedback.**

**Original message**

- Focus on something new by doing something hard. Try counting backward from 100 by sevens.

**Feedback**

- This feels silly and I wouldn’t do it.

**Revised message**

- Reduce your urge to use by focusing on something new and challenging.
- Try saying the alphabet backwards. Or, try coming up with as many words as you can that rhyme with “think.”

### Quantitative Results Following Revision

**Figure 2:**
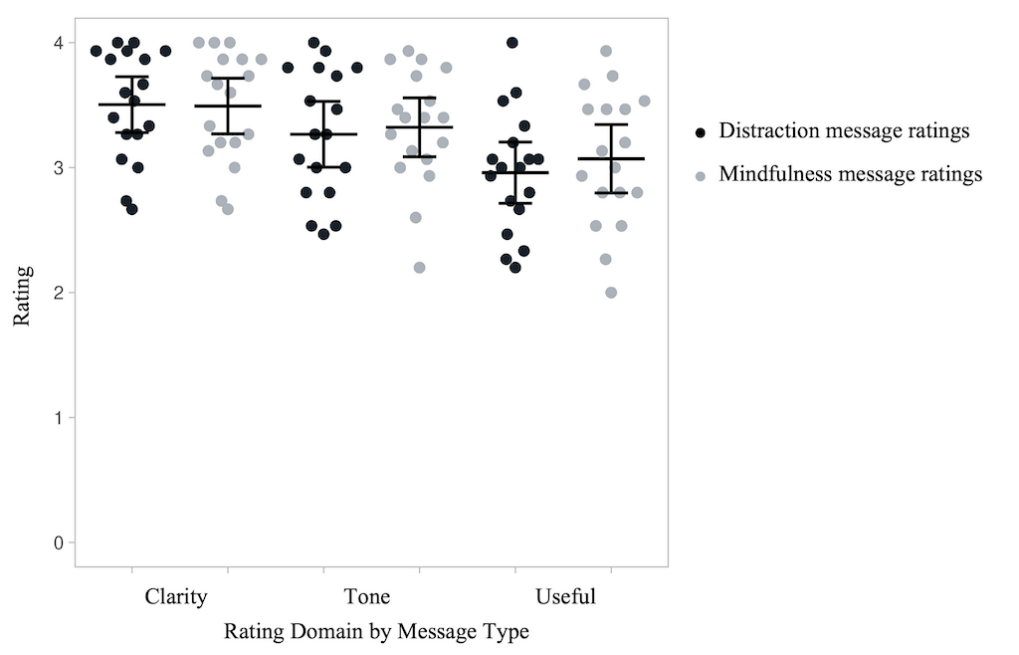
Participant ratings (n=18) of the distraction and mindfulness messages after the first round of message revisions. Each data point shows one participant’s average rating of the 15 distraction or 15 mindfulness messages on each of the three domains (clarity, tone, and usefulness). Horizontal bars show average ratings and error bars are 95% CIs.

We were unable to reach 2 participants to complete the second round of revisions. Therefore, 18 participants completed the second round of message evaluations. The 2 participants whom we could not reach were excluded from these analyses. Figure 2 shows the mean ratings for each category and message type following the second round of ratings. The ratings for the usefulness of the distraction messages improved significantly (t_17_=2.52, *P*=.02) with little change in clarity (t_17_=0.33, *P*=.75) or tone (t_17_=1.55 *P*=.14). Ratings for the tone of the mindful messages improved significantly (t_17_=2.64, *P*=.02) with very little change on clarity (t_17_=1.97, *P*=.07) and usefulness (t_17_*=*1.25, *P*=.23). All participants received the same number of messages to rate in the same order, and participants read and rated 1 message before the next message displayed. It is possible that the number of messages presented to the participants or the order in which the participants read the messages could have influenced the participants’ ratings. However, because the number of messages and the order in which they were presented were fixed, we cannot test the impact of order or number of messages on message satisfaction. Mean ratings for all items are in Table 2.

### Qualitative Results Following Revision

A number of themes emerged from the second round of feedback. For the mindfulness messages, the remaining concerns were primarily about some messages not being helpful (eg, 4 comments with concerns about the strategy not working or not being relatable), and there were some suggestions about how to reword messages to improve their clarity and usefulness (4 comments). For the distraction messages, the remaining concerns were primarily about the strategies not being helpful or practical (12 comments). We made final revisions to the messages based on the participants’ comments.

### Final Messages

**Figure 3:**
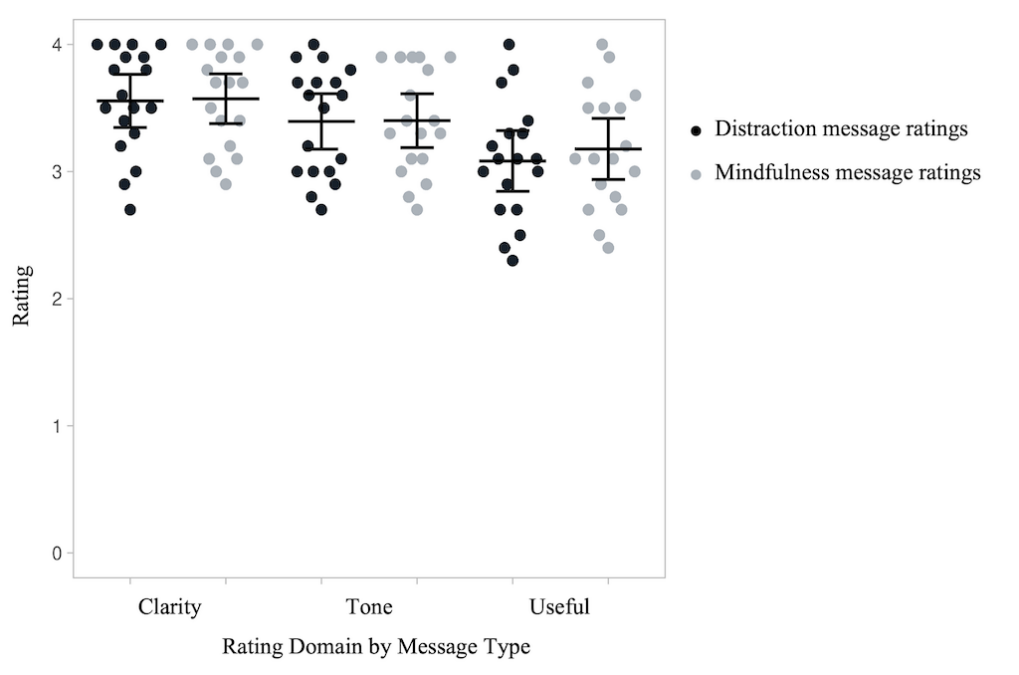
Participant ratings (n=18) of the final distraction and mindfulness messages after removing the unused messages. Each data point shows one participant’s average rating of the final 10 distraction or 10 mindfulness messages on each of the three domains (clarity, tone, and usefulness). Horizontal bars show average ratings and error bars are 95% CIs.

The top 10 messages of each coping type (mindfulness and distraction) were identified by overall (collapsed across all 3 dimensions) average rating. Figure 3 shows the mean ratings for each category and message type for the final messages selected. The final message items are in Multimedia Appendices 3 and 4. All final messages had an overall average rating ≥3.0 but selecting the top 10 messages allowed us to exclude messages that participants indicated may be triggering, suboptimal in tone, or particularly unhelpful from the pilot intervention. The second round of ratings of the final 20 distraction and mindfulness messages were not significantly different from each other in clarity (distraction mean 3.56, SD 0.41; mindfulness mean 3.57, SD 0.38; t_17_=0.25, *P*=.81), tone (distraction mean 3.39, SD 0.43; mindfulness mean 3.40, SD 0.41; t_17_=0.08, *P*=.94), and usefulness (distraction mean 3.08, SD 0.47; mindfulness mean 3.19, SD 0.47; t_17_=0.98, *P*=.34). These messages were used in a subsequent ecological momentary intervention study.

## Discussion

### Principal Findings

We set out to develop messages to be used in a pilot digital intervention to help young adults cope with cannabis cravings, including young adults who use cannabis in the message development process. This formative process resulted in changes to the original messages, and the selection of the highest-rated messages to be used in the subsequent intervention. Items improved significantly in terms of usefulness (distraction messages) and tone (mindfulness messages) following the first round of revisions. Although there were no significant improvements on the other domains following revisions, this was likely due to a ceiling effect, given the high initial ratings (mean >3.0 out of the maximum 4.0) on these domains. Initially, the participants found the distraction items to be clearer than the mindfulness messages, and the mindfulness messages to be more useful than the distraction messages. Following the revisions and final selection of 10 messages each from the mindfulness and distraction categories, the ratings of each message type were high and comparable across all domains.

### Comparison With Prior Work

Prior work on SMS text message intervention development has highlighted the importance of including end user populations in the development and design of the intervention [38,42]. Formative research to develop mobile health interventions has used focus groups and surveys to gain feedback from the target audience about elements of apps that participants find useful, to help prioritize various app features, and to identify barriers to implementation [43,44]. Additionally, previous studies developing text-based interventions have involved members of the target population in focus groups to develop message content and offered input on the timing and frequency of the SMS text messages [32,41]. Although focus groups and semistructured interviews may be particularly informative when there are multiple developmental components to consider such as design features and intervention content, our mobile intervention was developed for delivery via a commercial platform thus limiting the flexibility of app design.

Unlike other preliminary studies focused on text-based interventions, we did not conduct focus groups. Instead, we developed our initial messages based on existing evidence-based interventions, sought input from users on the content we developed, and modified our content based on participants’ feedback. Future research could implement a more comprehensive participatory approach in developing the various components of the intervention such as the timing and frequency of message delivery. One major finding of previous studies has been the importance of message tone on user engagement. The tone of the messages we developed is one domain on which our messages improved as a result of participant feedback, again highlighting the importance and benefits of including end users in the development of interventions.

### Limitations

The findings of this study should be considered in the context of a few limitations. We limited the involvement of our target population in developing the intervention to providing feedback on messages adapted from established interventions. This limitation is partially due to the constraints of the platform we used for our intervention delivery. The platform used allowed for flexible and diverse experimental designs and made the intervention possible at a low cost without the need for outsourcing programming but was limited in terms of app customization. After appropriate efficacy testing, it may be appropriate to distribute the app using a self-pay subscription model or by making the app freely available to consumers similar to other apps designed for those who are seeking assistance to moderate or abstain from substance use such as SoberTool developed by Blitzen, LLC. The costs of such apps can be sustained using in-app advertisements or possibly reimbursement through medical insurance [45].

A second limitation is regarding our sampling strategy. We limited our recruitment to social media in an attempt to reach a diverse population and allow users across the country to participate in the development of our intervention messages. This strategy allowed us to recruit nationwide and is the same planned strategy for recruiting our intervention participants. However, this method of recruitment may have resulted in bias due to self-selection. Additionally, due to time and monetary constraints, we solicited feedback from the participants using an asynchronous survey instead of more involved focus groups.

One additional limitation is that the user-rated usefulness of messages when craving levels are not currently elevated may be a poor proxy for clinical utility in the context of high craving; however, the usefulness of the messages will be evaluated in the subsequent intervention. We also did not distinguish between craving as a desire to use cannabis and urge as the intent to use cannabis. Although this is beyond the scope of this study, future studies may make this distinction and test whether 1 strategy (mindfulness or distraction) is better suited for managing the desire for the effects (ie, cravings) versus managing intent to use cannabis (ie, urges).

### Conclusions

The findings of this study support the importance and highlight the value of including the target intervention population in the formative process of intervention development. The content of the messages was significantly improved over the course of this formative process.

Additionally, the community identified possible triggers embedded in the messages that may have been counterproductive to our intervention—triggers that would not have been identified without their lived experience and inclusion in the message development. Focus groups may be more useful in developing and revising messages, allowing for additional conversation between the participants and the researchers, thus giving space for clarification and conversation. Sometimes, open-ended participant feedback comments were somewhat unclear, and having the opportunity to have an ongoing discourse could help develop messages further. The final messages developed in this study were subsequently used in a pilot intervention aiming to provide young adults who use cannabis with support for coping with their cannabis cravings as they attempt to reduce their use.

## Appendix

Multimedia Appendix 1 Initial bank of mindfulness messages.

Multimedia Appendix 2 Initial bank of distraction messages.

Multimedia Appendix 3 Final mindfulness messages.

Multimedia Appendix 4 Final distraction messages.

## Abbreviations

EMA: ecological momentary assessment
