# Supplementary material for: End User Participation in the Development of an Ecological Momentary Intervention to Improve Coping With Cannabis Cravings: Formative Study
Source: JMIR Form Res. 2022 Dec 15;6(12):e40139. doi: 10.2196/40139 (PMC9801264; doi:10.2196/40139)
Supplement: Multimedia Appendix 1 [file formative_v6i12e40139_app1.docx]

*Initial Bank of Mindfulness Messages*

|  | **Original Mindfulness Messages** |
| --- | --- |
| 1 | You may be thinking "I can’t stand this," or "this is unbearable and not worth the effort." Just notice these thoughts of distress, letting them come and go. Remember, you can feel the urge to use cannabis, but you don’t need to act on those feelings. |
| 2 | Sometimes, trying to get rid of thoughts about using cannabis can make those thoughts stronger and more difficult to bear. Take a moment to notice those thoughts without trying to change them or make them go away. Don’t fight these thoughts, and instead focus on how they make you feel. These feelings will fade with time. |
| 3 | Pay attention to any thoughts or sensations you may be feeling right now. Let all of your thoughts come into your attention and then gradually fade away. As you focus on these feelings, they might grow stronger. Image them like a wave, growing in intensity until it reaches its peak, then decreasing. Remember to allow your mind and body to feel anything they want. These are just sensations and they will pass. |
| 4 | Use this chance to practice staying with thoughts about using cannabis in a kind and curious way. You don’t need to act on your thoughts. Ask yourself: "What is it I truly need?" or "Is there a desire for relief?" Stay with this discomfort and explore it with gentle curiosity, knowing that it will dissipate with time. |
| 5 | If thoughts about using cannabis become intense, imagine yourself as a surfer riding a wave and using your breath to stay steady. Your job is to stay on top of the wave, through the peak of its intensity, until it naturally subsides. Watch this pattern as urges rise and fall. Trust that all waves or desire will fade away without any action on your part. |
| 6 | Stay present with your urges to use cannabis as they rise and fall like a wave. Stay present with this mindset and accept it without giving into it, acting upon it, or having to make it go away. Take a deep breath and try to move your body around a little. |
| 7 | Rather than fighting the urge to use cannabis, try to be curious about what you feel. Notice what you feel and watch the feelings pass by. |
| 8 | Notice any thoughts you have about your urge to use cannabis. Let these thoughts come into your attention, then just naturally fade away as though they were floating down a stream. |
| 9 | Notice whatever thoughts or sensations are happening right now and try letting these thoughts stay with you, rather than pushing them away. You do not need to change these thoughts or judge them as good or bad. |
| 10 | Remember, you can have whatever thoughts and feelings arise and still act differently than what you think or feel. Thoughts do not have to control action. |
| 11 | As you experience challenging feelings, try letting your thoughts stay with you. You don't need to change these thoughts. Instead, notice them and any discomfort they may bring without judgement. Pay attention to any sensations you may be experiencing and try to experience them as neither good nor bad but just events, as they rise and then pass. |
| 12 | Take the next minute to observe your urge to use. What does it feel like? Notice it without reacting to it by using cannabis. |
| 13 | Try “surfing the urge.” When you’re having a strong urge to use, watch as it feels more intense but eventually crashes, just like an ocean wave. Ride it out! |
| 14 | Take the next minute to observe your thoughts and feelings about using. What does it feel like? Notice your feelings without judgement, and without giving in to using cannabis. |
| 15 | Pay close attention to your thoughts and any physical sensations or changes in your body when you experience an urge to use cannabis. Feel your mouth watering, or your heartbeat speed up. Ask yourself, what does this feel like? Then let the feelings fade away. |
