# Supplementary material for: End User Participation in the Development of an Ecological Momentary Intervention to Improve Coping With Cannabis Cravings: Formative Study
Source: JMIR Form Res. 2022 Dec 15;6(12):e40139. doi: 10.2196/40139 (PMC9801264; doi:10.2196/40139)
Supplement: Multimedia Appendix 2 [file formative_v6i12e40139_app2.docx]

*Initial Bank of Distraction Messages*

| **Original Distraction Messages** | |
| --- | --- |
| 1 | We can only pay attention to so many things at one time. Try to distract yourself from thoughts about cannabis use by focusing on events or objects around you. You could look around the room and count all the round or rectangular objects. |
| 2 | You have more control over your feelings than you may think. Focus on something else to distract yourself from your urges to use cannabis. Listen to your favorite song and zero in on the lyrics and the beat. |
| 3 | The urge to use cannabis can feel overwhelming at times. Instead, focus on an activity you enjoy. This could be listening to music, watching a tv show, or going for a walk. Zero in on as much detail as you can! |
| 4 | Listen carefully to your surroundings. What do you hear? Birds outside? Cars? What about inside? Maybe you hear your air-conditioner or heater? Your computer humming? When we snap out of a focused state, it can be surprising to learn how much of the world around us we ‘tune out.’ This experience is one example of how controlling your attention can help you manage your thoughts about using cannabis. |
| 5 | Do you feel the ground under your feet, or the surface you’re sitting on? What about your socks or shoes? We don’t usually feel those things unless we focus on them. Try paying close attention to your body and notice the details that you typically ignore. |
| 6 | Imagine that you are in your favorite place. This can be a real place or imaginary. Try to see things in as much detail as possible, as though you are actually there. Involve all your senses. Think about what you see, hear, feel, and smell. |
| 7 | Go for a short walk either out in nature or just around your house - wherever you can get to right now. As you walk, pay close attention to your surroundings, what you can see, and what you can hear. |
| 8 | Distract yourself! Find the closest piece of furniture to you. Look at it carefully and think about how it was made. Consider all the steps that went into making this piece of furniture. If you’re outside, you could do this with a nearby building. Or, look closely at a plant and think about all the steps involved in its growth. |
| 9 | Focus your attention on something new! Look closely at an article of clothing. Can you pick out patterns? Try to count the stitches or imagine how it was made. |
| 10 | Make a plan for the future. Write out a to-do list for the day, or plan out your weekend activities. |
| 11 | With practice, distractions can become a great way to help you cope with your urges to use cannabis. Check out your Instagram feed, or find a funny filter on snapchat and send it to a friend! |
| 12 | Think of your attention as being like tv. You can focus your attention on only one channel at a time. If you have an urge to use cannabis, tune into another channel! Think about a place you want to go or imagine your favorite spot in as much detail as you can. |
| 13 | One way to distract yourself is to imagine something new in as much detail as possible. Imagine cutting and eating a lemon. What does the lemon look like? How does the lemon feel in your hands as you hold it? If you bring it up to your mouth, what does it smell like? How does it taste? Imagine this in as much detail as possible. |
| 14 | Focus on something new by doing something hard. Try counting backward from 100 by sevens. |
| 15 | Distract yourself with a challenge! Try to remember the lyrics to your favorite song without listening to the song. If that’s too easy, try choosing a different song! |
