# Supplementary material for: End User Participation in the Development of an Ecological Momentary Intervention to Improve Coping With Cannabis Cravings: Formative Study
Source: JMIR Form Res. 2022 Dec 15;6(12):e40139. doi: 10.2196/40139 (PMC9801264; doi:10.2196/40139)
Supplement: Multimedia Appendix 3 [file formative_v6i12e40139_app3.docx]

*Final Mindfulness Messages.*

| **Final Mindfulness Messages** | |
| --- | --- |
| 1 | You may be thinking "I can’t stand this," or "this is unbearable and not worth the effort." Notice these thoughts of distress without judgement. Remember, you don't need to act on any urges you may feel to use cannabis. |
| 2 | Sometimes, trying to get rid of thoughts about using cannabis can make those thoughts stronger and more difficult to bear. Take a moment to notice those thoughts without trying to change them or make them go away. Focus on how these thoughts make you feel instead of attempting to fight them. These feelings will fade with time. |
| 3 | Pay attention to any thoughts or sensations you may be feeling right now. As you focus on these feelings, they might grow stronger. Image them like a wave, growing in intensity until it reaches its peak, then decreasing. Remember to allow your mind and body to feel anything they want. These feelings will pass. |
| 4 | Use this chance to practice staying with thoughts about using cannabis in a kind and gentle way. You don’t need to act on your thoughts. Stay with this discomfort and explore it with gentle curiosity, knowing that it will dissipate with time. |
| 5 | If thoughts about using cannabis become intense, imagine yourself as a surfer riding a wave and using your breath to stay steady. Your job is to stay on top of the wave, through the peak of its intensity. Waves naturally subside - ride it through its peak and you've been successful. |
| 6 | Be present with your urges to use cannabis as they come and go. Stay with this mindset and accept it without giving into it, acting upon it, or having to make it go away. Take a deep breath. Before you know it, these feelings will pass. |
| 7 | Rather than fighting the urge to use cannabis, pay attention to what you feel without judgement. Watch the feelings pass by and trust that all desire will fade away without any action on your part. |
| 8 | Notice any thoughts you have about your urge to use cannabis and remember they are temporary. Let these thoughts come into your attention, then just naturally fade away as though they were floating downstream. Take deep breaths as your feelings fade. |
| 9 | Let whatever thoughts or sensations you're having right now stay with you. You do not need to push these thoughts away, change them, or judge them as good or bad. |
| 10 | Remember, you can have whatever thoughts and feelings arise and still act differently than what you think or feel. Thoughts do not have to control action. |
