# Supplementary material for: End User Participation in the Development of an Ecological Momentary Intervention to Improve Coping With Cannabis Cravings: Formative Study
Source: JMIR Form Res. 2022 Dec 15;6(12):e40139. doi: 10.2196/40139 (PMC9801264; doi:10.2196/40139)
Supplement: Multimedia Appendix 4 [file formative_v6i12e40139_app4.docx]

| **Final Distraction Messages** | |
| --- | --- |
| 1 | Try to distract yourself from thoughts about cannabis use by looking around the room and counting all the round or rectangular objects. Focusing on events or objects around you may help distract you from difficult thoughts because we can only pay attention to so many things at one time. |
| 2 | You have more control over your feelings than you may think. Focus on something positive to distract yourself from cravings. Think about your favorite song and try to tap out the beat on a table or your lap. |
| 3 | The urge to use cannabis can feel overwhelming at times. Instead, focus on a different activity you enjoy. This could be reading a book or magazine, or going for a walk. As you do these things, zero in on as much detail as you can to direct your attention away from your cravings! |
| 4 | Focus your attention on the sounds around you. What do you hear? Birds or cars outside? What about inside? Maybe you hear your air-conditioner or heater? Your computer humming? It can be surprising to learn how much of the world around us we ‘tune out.’ This experience is one example of how controlling your attention can help you manage your thoughts about using cannabis. |
| 5 | Do you feel the ground under your feet, or the surface you’re sitting on? What about your socks or shoes? We don’t usually feel those things unless we focus on them. Instead of focusing on your thoughts about using cannabis, take control of your attention and focus on your body. Notice the details that you typically ignore. |
| 6 | One way to distract yourself from an urge to use cannabis is to create an imaginary scene in your mind and see how many details you can notice. For example, imagine wading in a river. How is the sun reflecting off the water? Is the river flowing fast or slow? What does the water feel like? What does the air smell like? Imagine this in as much detail as possible. |
| 7 | Go for a short walk either out in nature or just around your house - wherever you can get to right now. As you walk, pay close attention to your surroundings, what you can see, and what you can hear. This can help distract you from thinking about cannabis. |
| 8 | Distract yourself from thoughts about using cannabis by focusing your attention on something else. Look at the nearest object and notice as much detail as you can. Do you see different patterns in fabric stitches or wood grain? If you’re outside, you could notice details in a nearby building. Or look closely at a plant and notice the different patterns in the leaves and the subtly different colors. |
| 9 | Instead of thinking about using cannabis, refocus your attention by doing something different! Suck on a piece of hard candy or chew some gum. Pay attention to how it tastes and how it feels in your mouth. |
| 10 | Make a plan for the future to distract yourself from what you're feeling right now. Write out a to-do list for the day or plan out your weekend activities. |

*Final Distraction Messages.*
